# Wild-Type Mitochondrial DNA Copy Number in Urinary Cells as a Useful Marker for Diagnosing Severity of the Mitochondrial Diseases

**DOI:** 10.1371/journal.pone.0067146

**Published:** 2013-06-27

**Authors:** Hui Liu, Yinan Ma, Fang Fang, Ying Zhang, Liping Zou, Yanling Yang, Sainan Zhu, Songtao Wang, Xuefei Zheng, Pei Pei, Lin Li, Hairong Wu, Yang Xiao, Yufeng Xu, Liwen Wang, Yanyan Cao, Hong Pan, Yu Qi

**Affiliations:** 1 Central Laboratory, Peking University First Hospital, Beijing, China; 2 Department of Neurology, Beijing Children’s Hospital, Beijing, China; 3 Department of Pediatrics, Beijing 301 Hospital, Beijing, China; 4 Department of Pediatrics, Peking University First Hospital, Beijing, China; 5 Department of Biostatistics, Peking University First Hospital, Beijing, China; 6 Department of Neurology, Capital Institute of Pediatrics, Beijing, China; University of Texas Health Science Center at San Antonio, United States of America

## Abstract

The genotype-phenotype relationship in diseases with mtDNA point mutations is still elusive. The maintenance of wild-type mtDNA copy number is essential to the normal mitochondrial oxidative function. This study examined the relationship between mtDNA copy number in blood and urine and disease severity of the patients harboring A3243G mutation. We recruited 115 A3243G patients, in which 28 were asymptomatic, 42 were oligo-symptomatic, and 45 were poly-symptomatic. Increase of total mtDNA copy number without correlation to the proportion of mutant mtDNA was found in the A3243G patients. Correlation analyses revealed that wild-type mtDNA copy number in urine was the most important factor correlated to disease severity, followed by proportion of mutant mtDNA in urine and proportion of mutant mtDNA in blood. Wild-type copy number in urine negatively correlated to the frequencies of several major symptoms including seizures, myopathy, learning disability, headache and stroke, but positively correlated to the frequencies of hearing loss and diabetes. Besides proportion of mutant mtDNA in urine, wild-type copy number in urine is also an important marker for disease severity of A3243G patients.

## Introduction

The A3243G mutation in mitochondrial DNA (mtDNA) is at a relatively high frequency of prevalence in population, and is associated with a wide spectrum of clinical manifestations [1], [2]. Mitochondrial diseases caused by this mutation are multi-system disorders and frequently involve the tissues with high energy demand, such as the nervous system, skeletal muscle, and myocardium. The proportion of mutant mtDNA is considered to be a determinant factor for the phenotype of the disease [3]. However, this conclusion is still in a matter of debate [4], [5]. Several studies have substantiated that the total mtDNA copy number plays a role in the phenotype of mitochondrial encephalomyopathies caused by mtDNA point mutations [6], [7]. Wild-type mtDNA copy number can be derived from the total mtDNA copy number and the proportion of mutant mtDNA. A recent study has shown that maintaining an optimal level of wild-type mtDNA plays a key role in retaining normal cytochrome C oxidase (COX) activity in segments of human skeletal muscle fibers harboring pathogenic mtDNA mutations [8]. So far, however, studies on the relationship between wild-type mtDNA copy number and phenotype of mitochondrial encephalomyopathies are unavailable. Here we performed a study to reveal wild-type mtDNA copy number in urine and blood in relation to disease severity and frequency of clinical symptoms in patients with A3243G mtDNA mutation.

## Subjects and Methods

### Ethics Statement

This study was approved by the Medical Ethics Committee of Peking University First Hospital. Informed written consent was obtained from the patients or their guardians and healthy controls.

### Patients

A total of 115 patients diagnosed to carry A3243G mutant mtDNA during the period from 2005 through 2012 and with the average age of 22 years old (0.5∼60 years old) were recruited from Pediatrics Department and Neurology Department of Peking University First Hospital, Beijing Children's Hospital, Capital Institute of Pediatrics, and Pediatrics Department of Beijing 301 Hospital. These patients were assigned based on disease severity into asymptomatic (no significant symptom/sign), oligo-symptomatic (only one symptom/sign) or poly-symptomatic (multiple symptoms/signs) group. In addition, 103 healthy subjects with the average age of 11 years old (1∼58 years old) were recruited as the healthy control group from the Physical Examination Center of Peking University First Hospital. Examinations for common point mutations and deletions in mtDNA in control’s peripheral blood and urine samples were negative, and physical and biochemistry examinations had been performed to exclude systemic diseases such as neurological diseases, hypertension, diabetes mellitus and hyperlipidemia.

### DNA Isolation

Total DNA was extracted from peripheral leukocytes by the method of Miller et al [9]. Urine sample collected in the early morning was centrifuged at 1,500 rpm for 10 min, and total DNA in urinary sediment was extracted by the silica method [10].

### Copy Number Measurement of Wild-type mtDNA and A3243G Mutant mtDNA in DNA Samples using Real-time Quantitative PCR (qPCR)

We used the method of amplification refractory mutation system (ARMS) to design two allele-specific primers [11]. The forward primers 5′-AGGGTTTGTTAAGATGGCTCA-3′ and 5′-AGGGTTTGTTAAGATGGCTCG-3′ (at nucleotide positions 3223–3243; Underlined nucleotides are different from the normal sequences to ensure the specificity) were used to specifically amplify wild-type mtDNA and A3243G mutant mtDNA, respectively. A reverse primer 5′-TGGCCATGGGTATGTTGTTA-3′ (at nucleotide position 3319–3300) and a TaqMan probe 5′-FAM-CCCGGTAATCGCATAAAACTTAAAACTTTACAGTCAGAG-TAMRA-3′ (at nucleotide position 3245–3283) combined with one of the allele specific forward primers were used for the quantification of wild-type or A3243G mutant mtDNA by qPCR. A fragment of 101 bp genomic DNA in the single copy nuclear gene *β-hemoglobin* was measured by qPCR as internal reference using the forward primer 5′-ACCTCAAGGGCACCTTTGC-3′, reverse primer 5′-AAAACATCAAGCGTCCCATAGAC-3′, and TaqMan probe 5′-FAM-CACTGTGACAAGCTGCACGTGGATCC-BHQ2–3′. Three recombinant plasmids that contain the target wild-type mtDNA, A3243G mutant mtDNA, or *β-hemoglobin* genomic DNA for qPCR were constructed as the copy number standards. The copy number in a purified plasmid solution can be derived from its molar concentration and Avogadro constant.

The PCR mixture contained 1×PCR buffer, 2.0 mM Mg^2+^, 5 ng DNA sample or a defined amount of copy number standard, 200 µM/each dNTPs, 0.5 µM/each primers, 0.2 µM TaqMan probe, 0.5 µl ROX dye (Invitrogen), and 1.25 units Taq DNA polymerase in a total volume of 25 µl. qPCR was run in an ABI Prism 7500 instrument with the thermo-cycling condition of 95°C for 10 min, and 45 cycles of 95°C for 15s and 63°C for 60s. Measurements were carried out in triplicate. Serial dilutions of the copy number standards were included in every qPCR assay to construct standard curves for wild-type mtDNA, A3243G mutant mtDNA and *β-hemoglobin* genomic DNA. If a sample was measured to contain >1,000,000 or <1,000 copies, a lower or a higher dilution of the DNA sample was measured again to fit the result in the range of the standard curve.

Based on the fact that one cell contained two copies of *β-hemoglobin* genes, wild-type mtDNA copy number per cell and total mtDNA copy number per cell in samples could be calculated. Result of mtDNA copy number per cell was shown in logarithmic value (lg). The proportion of A3243G mutant mtDNA was the copy number ratio of A3243G mutant mtDNA/total mtDNA. Alternatively, the proportion of the A3243G mutant mtDNA could be calculated using the formula: proportion of A3243G mutant mtDNA = 1/(1+1/2^△CT^), where △CT is C_T_^wild-type^ − C_T_^mutant^ [12].

### Statistical Analysis

Numerical values were presented as mean ± standard deviation (SD). Independent *t*-test was used to compare the mtDNA copy number per cell between patients and controls. One-way ANOVA and LSD test were used to compare mtDNA copy number per cell and proportion of A3243G mutant mtDNA among groups. Correlations between study variables and disease severity were analyzed using Spearman’s correlation. The frequencies of the clinical features in patients with different levels of wild-type mtDNA copy number in blood and urine were compared by χ2 analysis for a trend (χ^2^_TR_), and only clinical features with the frequency of >15% were analyzed further. The lg value at the 5th percentile of the wild-type mtDNA copy number per cell in control urine samples was set as the lower limit of normal level. The software package of SPSS 16.0 for Windows was used for statistical analyses. A value of *P*<0.05 was considered as statistically significant.

## Results

### 1. Accuracy of the Real-time qPCR Method for the Measurement of Wild-type and A3243G Mutant Copy Number in this Study

**Figure 1:**
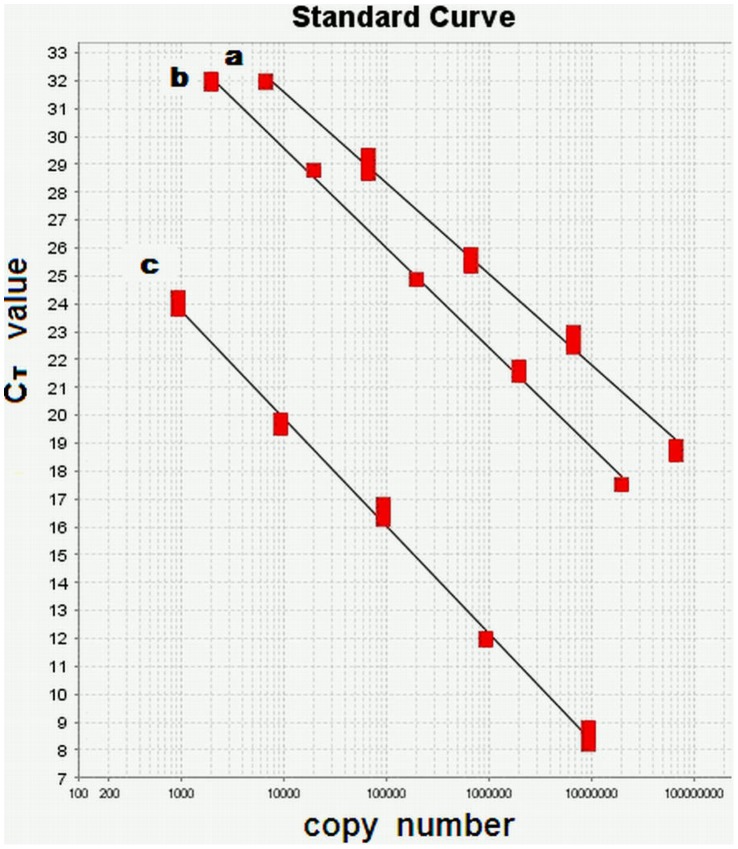
Standard curves for quantification of mtDNA. Copy number standards in 10-fold dilutions were used for qPCR, and the ranges of copy number used in this experiment were 6.77×10^7^∼6.77×10^3^ copies/µl for A3243G mutant mtDNA, 1.97×10^7^∼1.97×10^3^ copies/µl for wild-type mtDNA, and 9.53×10^6^∼9.53×10^2^ copies/µl for *β-hemoglobin* gene. (a) Standard curve for A3243G mutant mtDNA, slope = −3.257, r^2^ = 0.994; (b) standard curve for wild-type mtDNA, slope = −3.576, r^2^ = 0.998; (c) standard curve for *β-hemoglobin* gene, slope = −3.854, r^2^ = 0.996. Experiments were done in triplicate.

**Figure 2:**
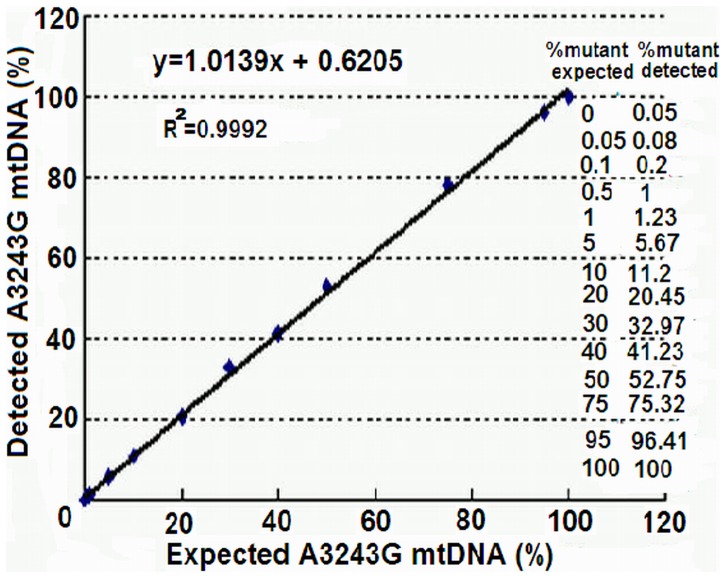
Accuracy of the qPCR for the measurement of wild-type mtDNA and A3243G mutant mtDNA. Copy number standards of wild-type mtDNA and A3243G mutant mtDNA were mixed in defined ratios and to a total copy number of 10^6^, and then subjected to qPCR to measure the ratios of A3243G mutant mtDNA. Results fit the expected ratios closely. Experiments were done in triplicate.

When 1,000,000∼1,000 copies of the copy number standards were used for qPCR, the primers for the amplification of wild-type mtDNA could not amplify the copy number standard of A3243G mutant mtDNA and *vice versa* (data not shown). Fig. 1 shows the linear standard curves of the 3 copy number standards over wider ranges of copy number, demonstrating that the errors due to sampling and manipulation of qPCR are acceptable. We then mixed copy number standards of wild-type mtDNA and A3243G mutant mtDNA in defined ratios to a total copy number of 10^6^, and measured the ratios of A3243G mutant mtDNA by qPCR. The correlation coefficient (R^2^) between detected ratios and expected ratios was 0.9992 (Fig. 2). For reproducibility of the measurements, we used a heteroplasmic A3243G mtDNA sample to measure A3243G mtDNA copy number in 6 replicates in one plate, and obtained the C_T_ value of 22.46±0.35 with intra-assay coefficient variation of 1.56%. We also measured A3243G mtDNA copy number in another heteroplasmic A3243G mtDNA sample in triplicate and for 6 times, and acquired the C_T_ value of 20.08±0.39 with inter-assay coefficient variation of 1.94%.

### 2. Wild-type Copy Number Per Cell in Urine was an Important Factor Correlating to Disease Severity of A3243G Patients

**Figure 3:**
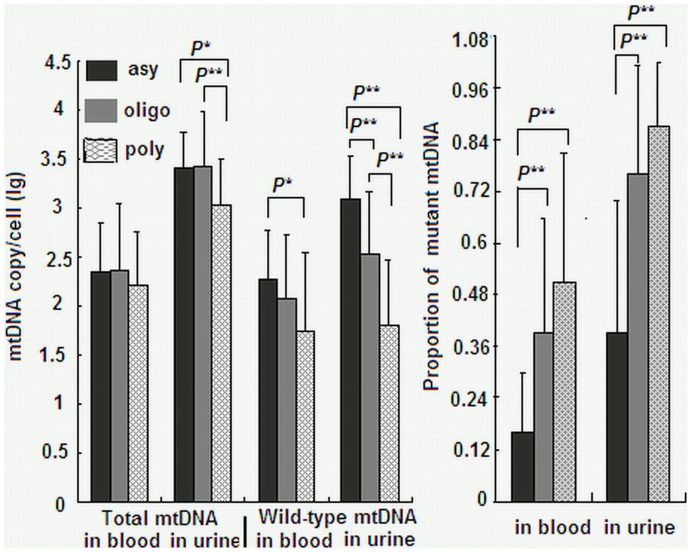
Results among asymptomatic, oligo-symptomatic and poly-symptomatic A3243G patients. Total mtDNA copy number per cell, wild-type mtDNA copy number per cell and proportion of mutant mtDNA in blood and urine in asymptomatic, oligo-symptomatic and poly-symptomatic A3243G patients. *: *P*<0.01; **: *P*<0.001. Every sample was measured in triplicate.

In the 115 A3243G patients, 28 were asymptomatic, 42 were oligo-symptomatic, and 45 were poly-symptomatic. The major manifestations in the 115 patients included seizure (67.7%), lactic acidosis (58.6%), myopathy (55.6%), learning disability (52.6%), headache (45.9%), hearing loss (35.3%) and diabetes (33.8%). Spearman’s correlation analyses found that wild-type mtDNA copy number in urine was the most important factor correlating to disease severity, followed by proportion of mutant mtDNA in urine and proportion of mutant mtDNA in blood (Table 1). Fig. 3 shows total mtDNA copy number, wild-type copy number and proportion of mutant mtDNA in asymptomatic, oligo-symptomatic and poly-symptomatic patients. Wild-type mtDNA copy number per cell in urine and in blood were closely correlated to disease severity (for urine: F = 40.406, *p<*0.001; for blood: F = 5.296, *p* = 0.006), with the lower copy number seen in severer patients. Likewise, proportion of mutant mtDNA in urine and in blood were closely correlated to disease severity (for urine: F = 38.074, *p<*0.001; for blood: F = 14.893, *p<*0.001), with the higher proportion of mutant mtDNA seen in severer patients. Among the three groups of A3243G patients, the difference in total mtDNA copy number in blood and urine was less prominent than that in wild-type copy number and proportion of mutant mtDNA in blood and urine (Fig. 3).

**Table 1:** Relationships between mtDNA copy number and disease severity in A3243G patients.

mtDNA copy number	r	*p*
Wild-type mtDNA copy/cell (urine)	−0.669	<0.001
Wild-type mtDNA copy/cell (blood)	−0.28	0.002
Proportion of mutant mtDNA (urine)	0.583	<0.001
Proportion of mutant mtDNA (blood)	0.455	<0.001
Total mtDNA copy/cell (urine)	−0.346	<0.001
Total mtDNA copy/cell (blood)	−0.135	0.152

### 3. Wild-type Copy Number Per Cell Correlated to the Frequencies of Several Major Symptoms in A3243G Patients

**Figure 4:**
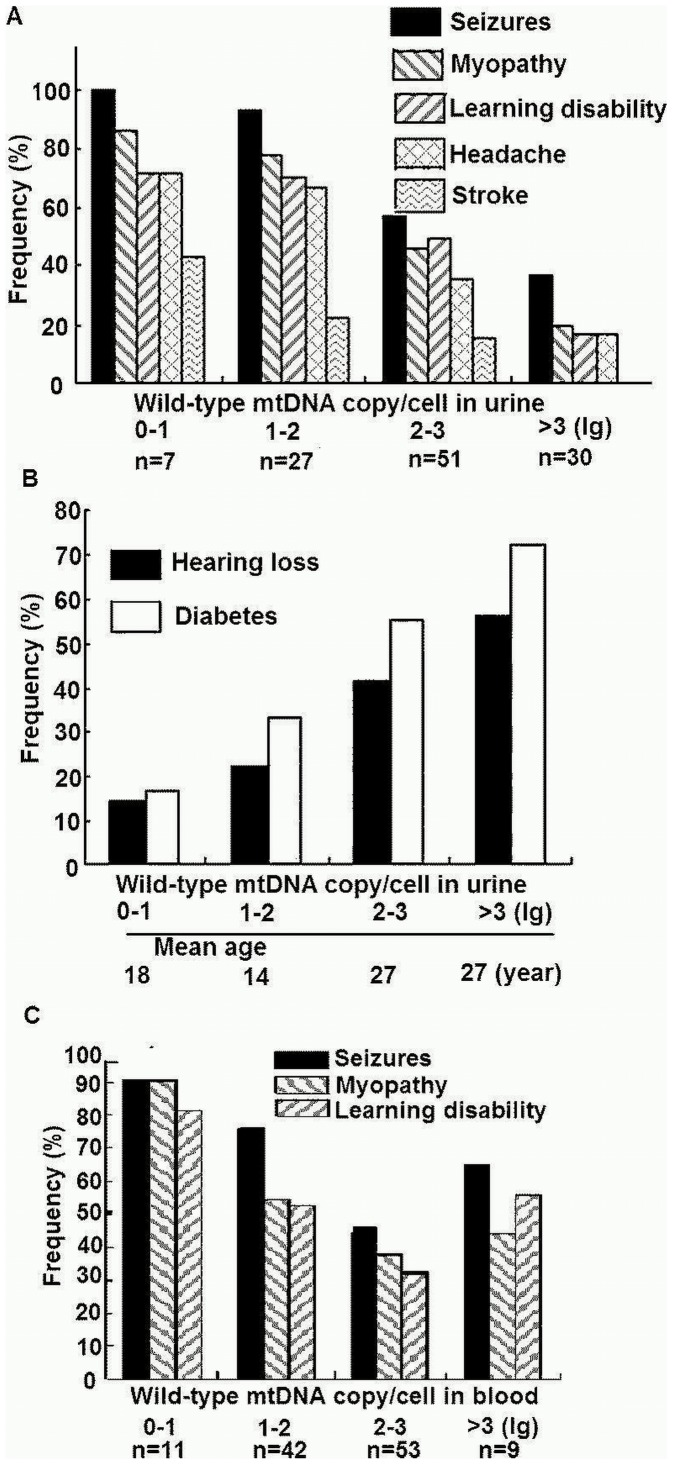
Relationship between the frequencies of major symptoms and the wild-type mtDNA copy number per cell in urine and blood. The 115 patients were divided into 4 subgroups based on the lg value of wild-type mtDNA copy number per cell in urine and in blood. Frequency of the major symptoms are shown in relation to the wild-type mtDNA copy number per cell in urine (panels A and B) and in blood (panel C).

When the 115 patients were divided into 4 subgroups based on the lg value of wild-type copy number per cell in urine, there were 7 patients with lg 0–1, 27 patients with lg 1–2, 51 patients with lg 2–3, and 30 patients with lg >3 (Fig. 4). Wild-type copy number per cell in urine was negatively correlated to the frequencies of seizures (χ^2^_TR_ = 22.485, *p*<0.001), myopathy (χ^2^_TR_ = 22.142, *p*<0.001), learning disability (χ^2^_TR_ = 16.819, *p*<0.001), headache (χ^2^_TR_ = 17.022, *p*<0.001), and stroke (χ^2^_TR_ = 13.484, *P*<0.001) (Fig. 4A), but was positively correlated to the frequencies of hearing loss (χ^2^_TR_ = 7.882, *P* = 0.005) and diabetes (χ^2^_TR_ = 8.712, *P* = 0.003) (Fig. 4B). Similarly, Wild-type copy number per cell in blood was negatively correlated to the frequencies of seizures (χ^2^_TR_ = 8.245, *p* = 0.004), myopathy (χ^2^_TR_ = 7.955, *p* = 0.005), and learning disability (χ^2^_TR_ = 5.411, *p* = 0.02) (Fig. 4C). No significant relationship was observed between wild-type copy number per cell in blood and the frequencies of stroke (χ^2^_TR_ = 1.257, *P* = 0.262), headache (χ^2^_TR_ = 3.388, *p* = 0.066), hearing loss (χ^2^_TR_ = 0.623, *P* = 0.43), and diabetes (χ^2^_TR_ = 1.827, *P* = 0.177).

### 4. Wild-type mtDNA Copy Number Per Cell as Well as Proportion of A3243G Mutant mtDNA in Urine Correlated to Disease Severity of A3243G Patients

**Figure 5:**
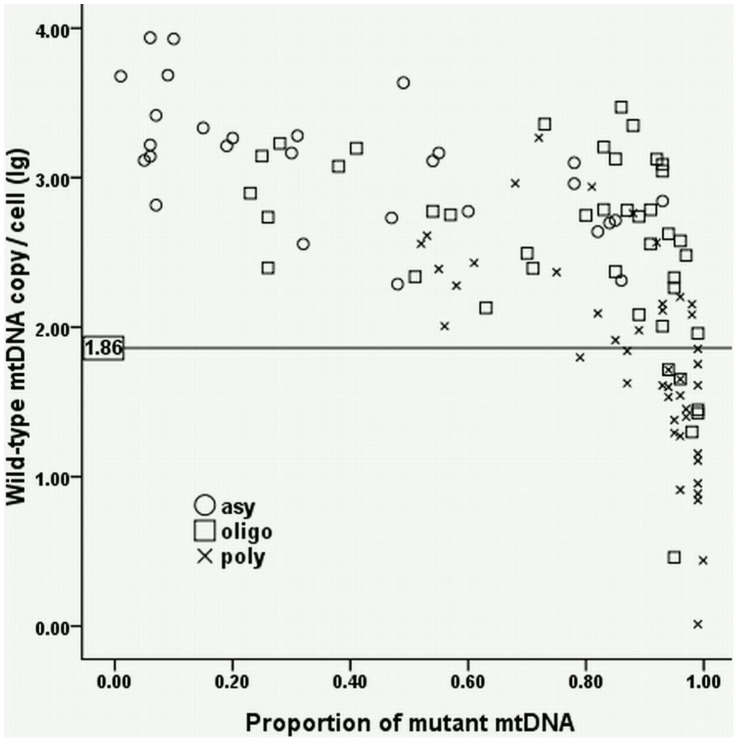
Relationship between wild-type mtDNA copy number and proportion of mutant mtDNA in urine. The lg value (1.86) at the 5th percentile of the wild-type mtDNA copy number per cell in control urine samples is set as the lower limit of normal level. 100%, 93%, and 44.4% of the patients locate above the limit in asymptomatic, oligo-symptomatic, and poly-symptomatic patients, respectively. For proportion of A3243G mutant mtDNA in urine, 39.3%, 83.3%, and 100% of the patients had the proportion of >0.5 in asymptomatic, oligo-symptomatic, and poly-symptomatic patients, respectively, and 77.7% of the poly-symptomatic patients carried the proportion of >0.8.

Fig. 5 shows the relationship between wild-type mtDNA copy number per cell and proportion of A3243G mutant mtDNA in urine in different severities of A3243G patients. In Fig. 5, 100%, 93%, and 44.4% of the patients locate above the 5th percentile of the control values in asymptomatic, oligo-symptomatic, and poly-symptomatic patients, respectively, additionally demonstrating the wild-type copy number per cell in urine closely correlated to disease severity of A3243G patients. Besides, proportion of A3243G mutant mtDNA in urine was also an important factor related to the disease severity. The proportion of >0.5 was found in 11 (39.3%) asymptomatic, 35 (83.3%) oligo-symptomatic, and 42 (100%) poly-symptomatic patients; the proportion of >0.8 was found in 5 (17.8%) asymptomatic, 25 (59.5%) oligo-symptomatic, and 35 (77.7%) poly-symptomatic patients. In contrast in blood, however, wild-type mtDNA copy number per cell and proportion of A3243G mutant mtDNA were correlated irregularly to the severity of the disease (data not shown).

### 5. Total mtDNA Copy Number Per Cell Increased in A3243G Patients

Total mtDNA copy number per cell was significantly higher in A3243G patients than in controls both in blood (t = −3.812, *p*<0. 001) and in urine (t = −3.882, *p*<0.001). If the patients and controls were divided based on age into 3 subgroups (0–10, 10–20 and >20 years of age), higher total mtDNA copy number per cell was still found in blood and urine in all 3 subgroups, but the total mtDNA copy number per cell in urine was statistically insignificant (t = −1.908, *p = *0.061) between patients and controls in the <10 age subgroups (Table 2). Total mtDNA copy number per cell was unrelated to the proportion of mutant mtDNA in blood (r = −0.096, *P* = 0.309) and in urine (r = 0.04, *P* = 0.67).

**Table 2:** Difference in total mtDNA copy number between A3243G patients and normal controls.

Age (year)	Normal controls		A3243G patients		
	n	mtDNA copy/cell mean ± SD (lg)	n	mtDNA copy/cell mean ± SD (lg)	*P*
0–10	30		33		
Blood		1.81±0.50		2.15±0.55	0.013
Urine		2.98±0.54		3.22±0.46	0.061
10–20	31		24		
Blood		2.18±0.47		2.57±0.56	0.007
Urine		3.38±0.49		3.66±0.48	0.041
>20	42		58		
Blood		2.11±0.34		2.30±0.59	0.045
Urine		2.65±0.61		3.14±0.48	<0.001
Total	103		115		
Blood		2.04±0.45		2.31±0.58	<0.001
Urine		2.97±0.63		3.27±0.51	<0.001

## Discussion

In this study, we found that wild-type mtDNA copy number in urine was the most important factor relating to disease severity, followed by proportion of mutant mtDNA in urine and proportion of mutant mtDNA in blood. Decrease of wild-type mtDNA copy number in urine and increase of proportion of mutant mtDNA in urine and blood were often associated with the increase of disease severity in A3243G patients, indicating that wild-type mtDNA copy number in urine is also an important marker for disease severity of A3243G patients. Additionally, total mtDNA copy number in blood and urine increased significantly in most A3243G patients, and lower total mtDNA copy number in urine was only seen in the severest patients.

A close relationship between frequencies of major symptoms of A3243G patients and wild-type mtDNA copy number was found both in urine and in blood. Lower wild-type mtDNA copy number in urine was often associated with the increased frequencies of several major symptoms (seizures, myopathy, learning disability, headache and stroke) in A3243G patients. Optimal wild-type mtDNA copy number is essential to maintain a normal oxidative function in organs especially in nervous system, skeletal muscle and myocardium with higher energy demand. Hence it is not surprising that the higher frequencies of central nervous system symptoms were usually present in A3243G patients with lower wild-type mtDNA copy number in urine. Interestingly, the frequencies of hearing loss and diabetes were higher in patients with higher wild-type mtDNA copy number in urine (Fig. 4B). The mechanisms underlying this apparent paradox are unclear, and age may be one of the determining factors in the development of these two symptoms in the presence of A3243G mutant mtDNA. As shown in Fig. 4B, patients with lower wild-type mtDNA copy number in urine had a lower mean age than those with higher wild-type mtDNA copy number in urine. Other studies have also highlighted that the mean age at the diagnosis of diabetes is 37±11 years [13], ranging from 11 to 68 years [14], [15]. The paradoxical findings suggest that patients with a lower wild-type mtDNA copy number in urine are likely to present central nervous system symptoms at a young age, whereas those with a higher wild-type mtDNA copy number in urine are liable to have hearing loss and diabetes in later years. Similarly, lower wild-type mtDNA copy number in blood was associated with the increased frequencies of the three major symptoms (seizures, myopathy and learning disability), but was statistically unrelated to the frequencies of headache, stroke, hearing loss and diabetes in A3243G patients. Therefore, both the wild-type mtDNA copy number in urine and in blood can be used to evaluate the disease severity, but wild-type mtDNA copy number in urine is preferable to be used to predict clinical severity in A3243G patients and their family members.

In our study, no definite relationship was observed between total mtDNA copy number and proportion of mutant mtDNA in blood and in urine, whereas wild-type mtDNA copy number correlated significantly to proportion of A3243G mutant mtDNA. The results may be consistent with the “maintenance of wild-type” hypothesis, which proposes that mutant mtDNA in cells triggers a compensatory mitochondrial proliferative response in order to maintain an optimal level of wild-type mtDNA genomes. However, when mutant amount reaches to a critical level, further nonselective mitochondrial proliferation leads to replication of the mutant species at the expense of wild-type mtDNA and eventually becomes detrimental to cellular survival [16], [17]. Recent study indicated that not only the amount of A3243G mutation but also the wild-type mtDNA copy number involved interference with normal functions [8]. We used the 5th percentile of the lg level of wild-type copy number in control urine samples as the lower limit of normal level, and found that wild-type copy number in urine was higher than this lower limit in all asymptomatic and most oligo-symptomatic patients, despite the higher mutation load in oligo-symptomatic patients than in asymptomatic patients. This result raises the possibility that higher mutation load leads to decompensation of wild-type mtDNA function followed by decrease of wild-type mtDNA copy number in urine. There are several biases in the analyses of results because of the variation of mtDNA copy number with age, but we believe that these biases will, at least in part, be counterbalanced by the sufficient number of normal controls (n = 103) and more than 30 patients and controls in every age subgroup. The mechanism of A3243G mutation interfering in the normal function of wild-type mtDNA remains unclear. A3243G mutation disturbs protein synthesis by decreasing ratio of aminoacyl-tRNA [Leu(UUR)] versus uncharged tRNA [Leu(UUR)] molecules and accumulating aminoacylation with leucine without any misacylation [18]. In the presence of defective tRNA, nuclear genes produce multi-copy suppressors such as cognate aminoacyl-tRNA synthetase, mitochondrial protein synthesis elongation factor and tRNA modification enzyme to interact with the mutated tRNA [19], [20]. However, the effect of these suppressors on the mutated tRNAs may be insufficient when the mutation load is high. Then wild-type mtDNA can no longer compensate for the effect of the mutation, leading to the impairment of mitochondrial function. Our previous study revealed that the proportion of A3243G mtDNA mutation in urine could reflect the disease severity of A3243G patients [21]. This study shows that wild-type mtDNA copy number in urine also closely relates to the disease severity of A3243G patients. Therefore, the examination of urine specimen can provide more useful information than blood in the research of A3243G mutation disease and other mitochondrial diseases.
